# ChatGPT Versus National Eligibility cum Entrance Test for Postgraduate (NEET PG)

**DOI:** 10.7759/cureus.63048

**Published:** 2024-06-24

**Authors:** Sam Paul, Sridar Govindaraj, Jerisha Jk

**Affiliations:** 1 General Surgery, St John's Medical College Hospital, Bengaluru, IND; 2 Surgical Gastroenterology and Laparoscopy, St John's Medical College Hospital, Bengaluru, IND; 3 Pediatrics and Neonatology, Christian Medical College Ludhiana, Ludhiana, IND

**Keywords:** neet, medical education, machine learning, artificial intelligence, chatgpt

## Abstract

Introduction

With both suspicion and excitement, artificial intelligence tools are being integrated into nearly every aspect of human existence, including medical sciences and medical education. The newest large language model (LLM) in the class of autoregressive language models is ChatGPT. While ChatGPT’s potential to revolutionize clinical practice and medical education is under investigation, further research is necessary to understand its strengths and limitations in this field comprehensively.

Methods

Two hundred National Eligibility cum Entrance Test for Postgraduate 2023 questions were gathered from various public education websites and individually entered into Microsoft Bing (GPT-4 Version 2.2.1). Microsoft Bing Chatbot is currently the only platform incorporating all of GPT-4’s multimodal features, including image recognition. The results were subsequently analyzed.

Results

Out of 200 questions, ChatGPT-4 answered 129 correctly. The most tested specialties were medicine (15%), obstetrics and gynecology (15%), general surgery (14%), and pathology (10%), respectively.

Conclusion

This study sheds light on how well the GPT-4 performs in addressing the NEET-PG entrance test. ChatGPT has potential as an adjunctive instrument within medical education and clinical settings. Its capacity to react intelligently and accurately in complicated clinical settings demonstrates its versatility.

## Introduction

The National Board of Examinations in Medical Sciences (NBEMS) was founded by the Indian government in 1975 to raise the quality of medical education by instituting rigorous, nationwide postgraduate standards for exams in contemporary medicine and leveraging the country’s already existing infrastructure to enhance capacity [1]. According to the National Medical Commission Act 2019, the National Eligibility cum Entrance Test for Postgraduate (NEET PG) is an eligibility-cum-ranking test that is required as the only entrance exam for admission to various postgraduate courses that are conducted by NBEMS. Around 200,517 students appeared for the exam in March 2023, and the competition only increases every year, with only bright minds able to crack it with good ranks [2].

Artificial intelligence (AI) refers to a software application that can mimic a context-sensitive response or a natural language interaction (like a chat) with a human user via messaging services, websites, or mobile applications [3]. The theoretical groundwork for the field currently known as AI was established by Norbert Wiener, Alan Turing, and Claude E. Shannon [4]. Applications and research domains such as corporate intelligence, finance, healthcare, visual identification, cybersecurity, and many more have shown the value of AI approaches [5]. Large language models (LLMs) have emerged as cutting-edge AI systems that can process and deliver text with coherent communication and generalize to multiple tasks [6]. With the recent release of ChatGPT from OpenAI, which integrates deep learning and language models built on the Generative Pre-training Transformer (GPT) architecture, chatbot capabilities have been greatly expanded [7].

Because ChatGPT is versatile and all-purpose, its public release in November 2022 has caused a stir. Based on the GPT-3.5 architecture, ChatGPT has gained popularity because of its exceptional capacity to provide replies that are both logical and human-like. The most recent version, GPT-4.0, has better multi-turn conversation management and improved language creation [8]. Exams with multiple choice questions (MCQs) are used in medical education to assess students’ knowledge in various subject areas [9]. MCQs are a commonly utilized and dependable evaluation method in a variety of undergraduate and graduate medical exams [9]. Several studies have examined ChatGPT’s effectiveness in multiple choice tests within higher education fields. Writers have investigated ChatGPT’s proficiency in the medical domain by analyzing its performance on the United States Medical Licensing Examination, Ophthalmic Knowledge Assessment Program exam, United Kingdom Medical Licensing Assessment (UKMLA), and NEET UG [8,10-12]. However, as far as we are aware, no recent study has evaluated ChatGPT’s performance in the NEET PG exam.

NEET PG consists of 200 MCQs, with each question carrying 4 marks for the correct answer and -1 deduction for the incorrect answer [13]. There are no marks for unanswered questions. Preclinical, clinical, and paraclinical disciplines included in the MBBS program serve as the foundation for the NEET PG syllabus. Candidates are required to select the correct, best, or most appropriate response or answer out of the four response options provided in each question, and the time allotted is three hours and 30 minutes [13].

This study aims to assess ChatGPT’s performance on the NEET PG test. This may be used to gauge ChatGPT’s present level of clinical expertise and determine whether or not it is a trustworthy AI system that will support human learning in medical education.

## Materials and methods

This study was conducted at St John's Medical College Hospital, Bengaluru, India. For a variety of reasons, the NBEMS does not formally distribute the NEET PG questions and answer key. As a result, the NEET PG 2023 questions were gathered from various public education websites. We cross-checked each question’s response using literature from each topic area of expertise. There were 200 questions in all, some of which were situation-based on images. A small number of questions, mostly based on clinical settings, were simplified to aid with comprehension.

Two hundred NEET PG-2023 questions were gathered from various public education websites and individually entered into Microsoft Bing (GPT-4 Version 2.2.1). Microsoft Bing Chatbot is currently the only platform incorporating all of GPT-4’s multimodal features, including image recognition. Additionally, the questions were formatted more simply so that the AI bot could grasp them better. The results were subsequently analyzed. It provided one of the four options (A, B, C, or D) for each question, along with a justification for why it was the right response. For every submission, a new ChatGPT conversation session was started to lessen memory recall bias. GPT-4 had a single attempt for every question, including image-based ones. We never had the option of teaching or prompting the AI with further questions, as this would allow bias. The responses produced by GPT-4 for the NEET PG 2023 test questions were entered into a Microsoft Excel spreadsheet (Microsoft Corporation, Redmond, United States) after each attempt. Following that, the produced and the created answer keys were compared. As there are 19 subjects in the undergraduate curriculum, each subject’s performance was assessed.

## Results

Out of 200 questions, ChatGPT-4 answered 129 correctly, earning 445 marks, which is more than the 50th percentile needed to pass the test. For the same, the rank would have been in the range of 20,000 to 25,000. The most tested specialties were medicine (15%), obstetrics and gynecology (15%), general surgery (14%), and pathology (10%), respectively. A total of 60% of the questions were clinical scenarios, while 15-25% of the paper consisted of one-liner questions; 17.5% were image-based. GPT-4 answered 62.9% of image-based questions correctly, had a 100% strike rate on one-liner questions, and was unsuccessful in 39.2% when clinical scenarios were presented. The results are presented in Table 1.

**Table 1:** ChatGPT’s performance across subspecialties

Subject	Questions	Correct	Percentage correct
Surgery, orthopedics, anesthesia, and radiology	35	23	65.7%
Medicine, dermatology, and psychiatry	35	21	60%
Obstetrics and gynecology	30	18	60%
Pathology	20	11	55%
Pharmacology	20	15	75%
Social and preventive medicine	17	12	70.5%
Microbiology	15	9	60%
Pediatrics	6	4	66.6%
Ear, nose, and throat	4	3	75%
Ophthalmology	8	6	75%
Forensic medicine	5	4	80%
Anatomy, physiology, and biochemistry	5	3	60%

## Discussion

This study evaluated ChatGPT-4’s performance in NEET PG. Additionally, our results demonstrated ChatGPT-4’s ability to respond to image-based queries. Numerous studies have assessed ChatGPT’s performance on various medical exams. The most important conclusion of our research is the level of performance attained by ChatGPT in one of the most difficult examinations in India, the NEET PG, which doctors must pass to advance into specialization. According to our research, ChatGPT-4 performed reasonably well in NEET PG 2023. Furthermore, we did not teach or prompt the AI with any questions. In contrast to other research that offered many chances for each question, our study only allowed one attempt at the GPT-4. More model interaction and prompting would have frequently resulted in more accurate findings. Our study is among the few that assessed GPT-4’s capacity to respond to image-based questions as well.

Overall, in our study, performance was best for questions requiring basic knowledge and for clinical questions with clear and instructive substance. Because of the lengthy word count and unclear circumstances, GPT-4 performed poorly in clinical scenarios. As a result, the success rate for long, complicated questions was lower than that of short, basic ones, indicating that it is comparatively ineffective in human abilities like data processing and analysis, which is a similar finding to another study [14]. Crucially, as mentioned by Jiao et al. [15], GPT-4.0 demonstrated strength in answering image-related questions in our study. The length of GPT-4 answers is often correlated with the word count of the questions, with answers to questions that were answered poorly being much longer, which is similar to a study by Taloni et al. [16]. It was also noted that in our study, excessive verbosity was shown to be frequently linked to evasive and generic answers, leading to incorrect responses. In contrast to a recent study that showed a significant improvement in GPT-4’s performance when compared to both its predecessor and human volunteers, our investigation revealed that GPT-4 performed worse than humans [17].

In terms of the proportion of MCQs being answered correctly across different specialties, ChatGPT performed best in forensic medicine (80%), ear, nose, throat, ophthalmology, and pharmacology (75% in each). Its performance in pathology (55%) was the worst in our study. These results run counter to a study done by Wang et al. [17]. One possible explanation for the low pathology score is a lack of diversity in the data. An AI model may not be able to correctly identify pathology in a variety of patients if the dataset used to train it is not varied [18]. In the research, the Bing Chat bot answered all or most of the CT and X-ray questions that were posed to it accurately; however, for more complex questions, replies tended to err on the side of caution, resembling a study by Kuckelman et al. [19]. Jang and Lukasiewicz’s study [20] discovered that when input material is paraphrased, the resultant responses are inconsistent. As of now, no research has been done on the consistency of responses provided by ChatGPT in the context of medical education. We are unable to comment on the consistency of the responses provided by GPT-4 in our study because each question only received one attempt. Lai et al.’s [12] study found that there was inconsistency between GPT-4 and UKMLA test responses. However, there is insufficient research to draw the same conclusions for NEET PG. The artificial hallucinations might be the cause of the inconsistent responses from GPT-4 or other big language models [21].

Although we believe LLMs like ChatGPT will significantly influence medical information processing, they should be closely examined as emerging technologies. GPT-4’s response error rate makes it necessary to carefully consider its uses and associated hazards, especially in high-stakes medical circumstances. Using AI technologies as supplemental resources, medical professionals must continue to rely on their education, experience, and intuition. ChatGPT is rejected by many scientific publications and professionals since it lacks critical thinking skills and displays information in an illogical and repetitious manner [22]. According to Wang’s research [23], ChatGPT did a good job of responding to inquiries on foundational medical knowledge. It did, however, score badly on clinical questions that tested clinical reasoning and thinking abilities, such as case analysis and treatment option selection, which is in line with our results too.

Our study has various limitations, so it should be evaluated with caution. Several constraints need to be taken into account in this study. First, memory recall bias may have resulted from the fact that NEET PG questions are not publicly available and were created solely based on unique memories from diverse educational platforms. Second, because the GPT-4 was only allowed one opportunity to respond without prompting, it was not possible to confirm that it would always deliver the same answer.

## Conclusions

This study sheds light on how well the GPT-4 performs in addressing the NEET-PG entrance test. While success rates in many specializations were remarkable, there were noticeable inadequacies in several fields, such as pathology and medicine. The AI’s performance will increase as the huge language model learns more, as seen by the recent acceleration of advancement. Accepting ChatGPT’s limitations and possible misuse will be essential to the technology’s effective adoption in the healthcare industry.
